# Mutation of Serine 1333 in the ATR HEAT Repeats Creates a Hyperactive Kinase

**DOI:** 10.1371/journal.pone.0099397

**Published:** 2014-06-05

**Authors:** Jessica W. Luzwick, Edward A. Nam, Runxiang Zhao, David Cortez

**Affiliations:** Department of Biochemistry, Vanderbilt University School of Medicine, Nashville, Tennessee, United States of America; University of Oxford, United Kingdom

## Abstract

Subcellular localization, protein interactions, and post-translational modifications regulate the DNA damage response kinases ATR, ATM, and DNA-PK. During an analysis of putative ATR phosphorylation sites, we found that a single mutation at S1333 creates a hyperactive kinase. *In vitro* and in cells, mutation of S1333 to alanine (S1333A-ATR) causes elevated levels of kinase activity with and without the addition of the protein activator TOPBP1. S1333 mutations to glycine, arginine, or lysine also create a hyperactive kinase, while mutation to aspartic acid decreases ATR activity. S1333A-ATR maintains the G_2_ checkpoint and promotes completion of DNA replication after transient exposure to replication stress but the less active kinase, S1333D-ATR, has modest defects in both of these functions. While we find no evidence that S1333 is phosphorylated in cultured cells, our data indicate that small changes in the HEAT repeats can have large effects on kinase activity. These mutants may serve as useful tools for future studies of the ATR pathway.

## Introduction

Nucleotide imbalances, hard to replicate DNA sequences, and damage to the template strand create challenges for complete and accurate DNA replication. The replication stress response maintains genome integrity through sensing and overcoming these challenges by promoting the repair of the damaged DNA, stabilizing stalled replication forks, and activating cell cycle checkpoints [1]. The PI3K-related protein kinases (PIKKs), including ATM and Rad3-related (ATR), are primary regulators of the replication stress response [2].

PIKK kinases are large proteins with significant sequence homology and shared domain architecture. The N-terminus of these proteins consist of dozens of Huntington, Elongation factor 3, Protein phosphatase 2A, and PI3K TOR1 (HEAT) repeats; each containing two interacting anti-parallel alpha-helices connected by a flexible loop [3]. The kinase domain is located at the C-terminus and is flanked by the FRAP, ATM, TRRAP (FAT) domain [4], the PIKK regulatory domain (PRD) [5], and FAT C-terminus (FATC) domain [6]. The PIKKs preferentially phosphorylate serine or threonine residues followed by a glutamine (S/TQ), giving these kinases many overlapping substrates.

PIKK family members promote repair of different types of damaged DNA [7]. Ataxia-telangiectasia mutated (ATM) is activated by DNA double strand breaks, but ATR signals in response to a variety of DNA lesions, including double strand breaks, base adducts, and crosslinks. The common feature of these lesions is the generation of single stranded DNA either directly or as a consequence of enzymatic processing. Unlike ATM, ATR is essential for the viability of replicating human and mouse cells and is activated every S-phase to regulate replication origin firing, repair stalled replication forks, and prevent early entry into mitosis [8]–[12]. Rare, hypomorphic mutations in ATR are associated with Seckel syndrome, a disorder characterized by microcephaly, growth retardation, and other developmental problems [13]. Cancer cells have an increased dependence on the ATR pathway due to high levels of oncogene-induced replication stress and frequent loss of the G_1_ checkpoint [14]–[16]. This dependence makes the ATR pathway a promising cancer therapeutic target.

Generation of single stranded DNA gaps initiates ATR activation, which involves recruitment of a signaling complex containing multiple proteins including ATR, ATR-interacting protein (ATRIP), RAD9-HUS1-RAD1, and BRCT repeat protein topoisomerase binding protein 1 (TOPBP1) to the stalled fork [17]. This recruitment is largely mediated by the single-stranded DNA binding protein, replication protein A (RPA). TOPBP1 binds to the ATR-ATRIP complex promoting a conformational change that likely increases its affinity towards substrates [18], [19]. Subcellular localization to specific DNA lesions and additional protein activators are key regulatory elements for the PIKK family members.

Additionally, PIKKs are regulated by post-translational modifications. ATM auto-phosphorylation induces the transition from an inactive dimer to an active monomer [20]. Several ATR auto-phosphorylation sites have been identified, including threonine 1989 [21], [22]. However, T1989 is not evolutionarily conserved and there are conflicting data about how important its phosphorylation is to the ATR activation process [21], [22]. Finally, several other proteins have been suggested to regulate ATR activation, but their precise roles may be dependent on the type of initiating signal.

In the process of studying how ATR phosphorylation regulates its activity, we discovered that a single mutation at serine 1333 creates a hyperactive kinase. Both the basal activity level and TOPBP1-stimulated activity of the S1333A protein are significantly increased compared to the wild type protein. Additionally, S1333 mutations to glycine, arginine, or lysine also create hyperactive kinases. Conversely, a S1333D mutation decreases ATR activity. While we find no evidence that S1333 is phosphorylated in cultured cells, our studies indicate that mutation of a single serine in the large, HEAT repeat region of this 2,644 amino acid protein is sufficient to greatly alter its activity. The exact mechanism mediating this change will require a high-resolution structural analysis; however, these mutants provide useful tools for studying the ATR pathway.

## Materials and Methods

### Cell Lines

All cell lines were obtained from ATCC. HEK293T cells were maintained in DMEM +7.5% FBS. HCT116 ATR^flox/−^TR cells were generated previously [5], and maintained in McCoy’s 5A medium with 10% FBS and 10 µg/ml blasticidin. Stable clonal ATR cell lines with tetracycline inducible ATR cDNAs containing the FLAG-HA_3_ epitope-tag were generated as previously described [21], and maintained in McCoy’s 5A medium containing 10%FBS, 300 µg/ml hygromycin B, and 10 µg/ml blasticidin. Exogenous ATR expression was induced with 1 µg/ml tetracycline. Cre excision of the floxed allele was done as previously described [8]. PCR genotyping was done with the following primers to confirm excision of the floxed allele as previously described [8]: GTCTACCACTGGCATAACAGC and CAGCGGGAGCAGGCATTTC.

### DNA Constructs, Sequence Alignment, Structure Prediction

Site directed mutagenesis of ATR in a modified pCDNA5/TO FLAG-HA_3_ or pCDNA5/TO FLAG backbone was performed as previously described [21]. Sequence alignments utilized ClustalW2 [23]. The protein structure prediction was done with Phyre2 using ATR amino acids 1328–1364 for HEAT repeat 27 [24].

### Drug Treatment

Hydroxyurea (HU) was added at 0.2, 0.5, 1.0, or 2.0 mM as indicated. Ultraviolet C radiation (UV) was administered at 20 or 50 J/m^2^. Ionizing radiation (IR) was from a Cs^137^ source at a rate of 1.8 Gy/min, and cells were treated with 8 Gy.

### Mass Spectrometry

FLAG-ATR was immunopurified from transiently expressing HEK293T cells with anti-FLAG M2 beads (Sigma). ATR was eluted from the beads using FLAG peptide and then precipitated using trichloroacetic acid. Eluted protein was digested with trypsin or chymotrypsin and the resulting peptides were analyzed as previously described [25].

### *In vitro* Kinase Assays

Kinase assays were performed as previously described [5], [26]. Briefly, ATR-ATRIP complexes were isolated from HEK293T cells transfected with FLAG-ATR and HA-ATRIP expression vectors using anti-HA beads (Sigma). After purification, recombinant GST-TOPBP1-ATR activation domain (AAD) protein was added along with GST-MCM2 substrate, and [γ-^32^P]ATP. Reactions were separated by SDS-PAGE and ^32^P incorporation onto the substrates was measured by a phosphorimager. Fold activation was calculated by dividing [^32^P]-MCM2 intensity by the intensity of [^32^P]-MCM2 for non-activated wild-type ATR. Experiments were completed at least three times, and the figures present a representative experiment.

### Flow Cytometry

The HU and UV recovery and G_2_ checkpoint assays were completed as previously described [5], [27], [28].

### Western Blotting and Immunoprecipitations

Cell lysates were produced using Igepal detergent lysis buffer (1% Igepal CA630, 200 mM NaCl, 50 mM Tris (pH 8.0)). Co-immunoprecipitation of the ATR-ATRIP complex was done using nuclear extracts prepared by hypotonic swelling, dounce homogenization, and high salt extraction. Anti-FLAG M2 beads were washed three times with TGN buffer (50 mM Tris pH 7.5, 150 mM NaCl, 10% glycerol, 1% Tween 20, 0.2 mM phenylmethylsulfonyl fluoride, 0.5 mM dithiothreitol, 1 mM NaF, 1 mM sodium vanadate, 10 mM β-glycerol phosphate, 5 µg/mL aprotinin, and 5 µg/mL leupeptin) and once with TGN buffer containing 0.5 M LiCl.

Antibodies used include ATR-N19 (Santa Cruz Biotechnology), HA (Covance), CHK1-G4 (Santa Cruz Biotechnology), FLAG-M2 (Sigma), ATRIP403 [8], MCM2 (BD Transduction Labs), phosphorylated Ser-317 CHK1 (Cell Signaling Technology), phosphorylated Ser-345 CHK1 (Cell Signaling Technology), and phosphorylated Ser-10 Histone H3 (Cell Signaling Technology). Phosphorylated Ser-108 MCM2 antibody was described previously [29]. The phosphorylated Thr-1989 ATR antibody was generated by Epitomics with the following peptide antigen: cFPENEpTPPEGKNML. Quantitative immunoblotting was done with the Li-Cor Odyssey infrared imaging system. The values were typically measured for both the phosphorylated protein and the total protein and a ratio calculated to normalize for loading on the western blot. In addition, these ratios were then typically normalized to a single reference sample set at 1.0.

## Results

### Mutation of Serine 1333 Alters ATR Kinase Activity

**Figure 1:**
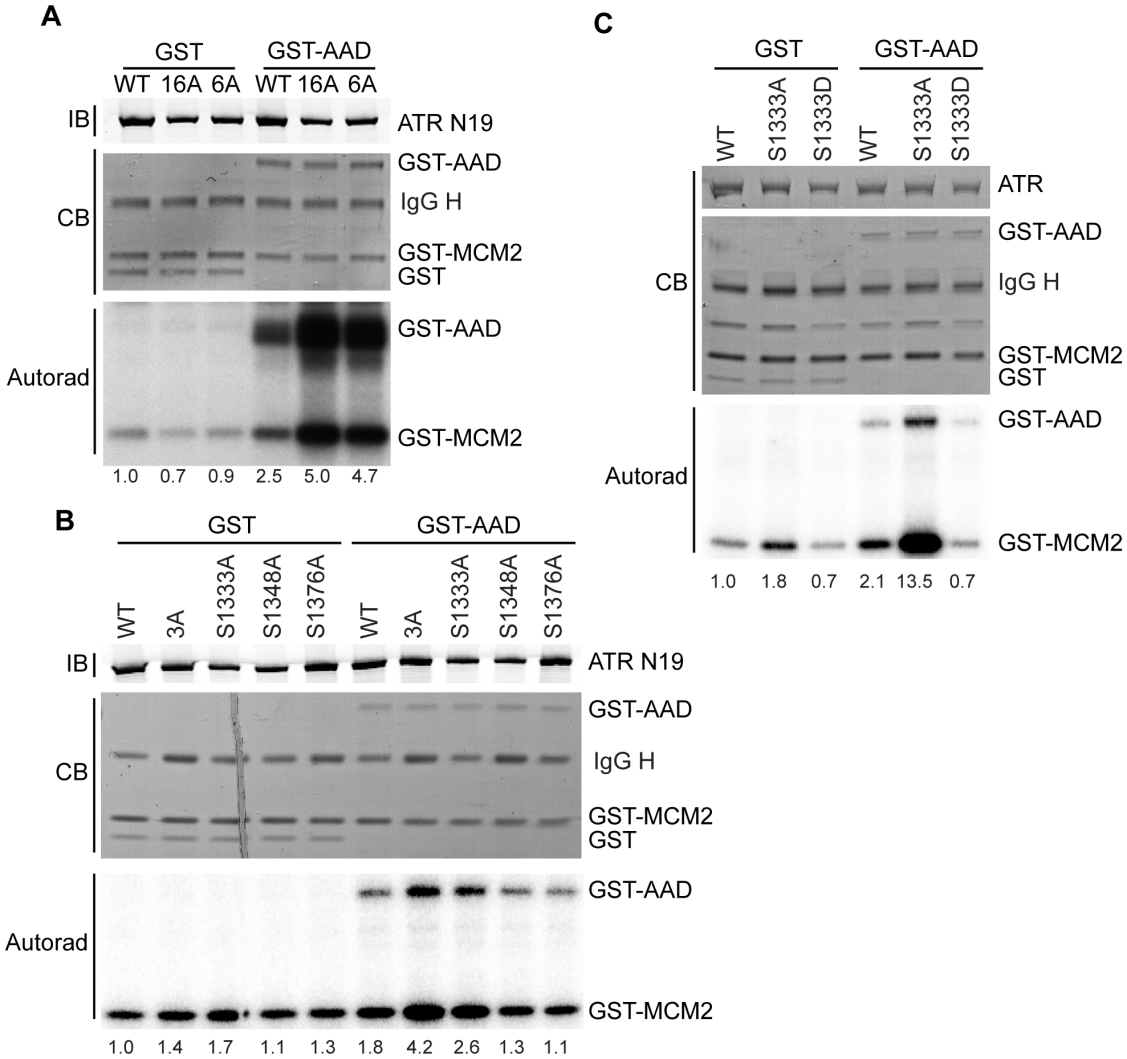
Mutation of S1333 to alanine creates a hyperactive ATR kinase. (A–C) The indicated ATR mutant or wild type proteins complexed with ATRIP were purified from HEK293T cells and incubated with GST-MCM2 substrate, [γ-^32^P]-ATP, and GST-TOPBP1-AAD (GST-AAD) or GST. Kinase reactions were separated by SDS-PAGE and [^32^P]-MCM2 and [^32^P]-GST-TOPBP1 detected by autoradiography. Quantitation of the fold activation compared to the wild type protein incubated with GST was measured by a phosphorimager and is indicated below each lane. The amount of ATR, TOPBP1, MCM2, and GST proteins in each reaction was detected with Coomassie blue (CB) or immunoblotting (IB) as indicated. Each mutant was tested at least three times and representative experiments are shown.

ATR preferentially phosphorylates S/TQs. ATR contains 19 of these putative phosphorylation sites. Sixteen of them are conserved in mice. To identify which of these serines may be functionally important, we mutated all sixteen conserved S/TQs to alanine within one cDNA. We then tested the kinase activity of the 16A-ATR protein using an *in vitro* kinase assay. The 16A-ATR mutations create a hyperactive kinase compared to wild type in kinase assays containing the AAD of TOPBP1 (Fig. 1A, [28]). Even when considerably less of the 16A-ATR was purified and added to the reaction compared to the wild type protein, it had significantly higher activity levels.

To determine which of the mutations in the 16A protein caused this hyperactivity, we tested a series of ATR proteins with subsets of these mutations. A 6A-ATR protein (S1333/S1348/T1376/S1782/T1890/S2143A) retained the elevated activity (Fig. 1A). The small difference between the 16A and 6A activities seen in this representative experiment is not reproducible. We further narrowed the relevant mutations to a 3A-ATR (S1333/S1348/S1376A) protein (Fig. 1B). Finally, a single alanine mutation, revealed S1333A as the primary mutation inducing the hyperactivity (Fig. 1B). The small difference between the S1333A and 3A protein activities in this experiment is due to the reduced amount of 3A protein purified and was not observed in replicate experiments (data not shown).

**Figure 2:**
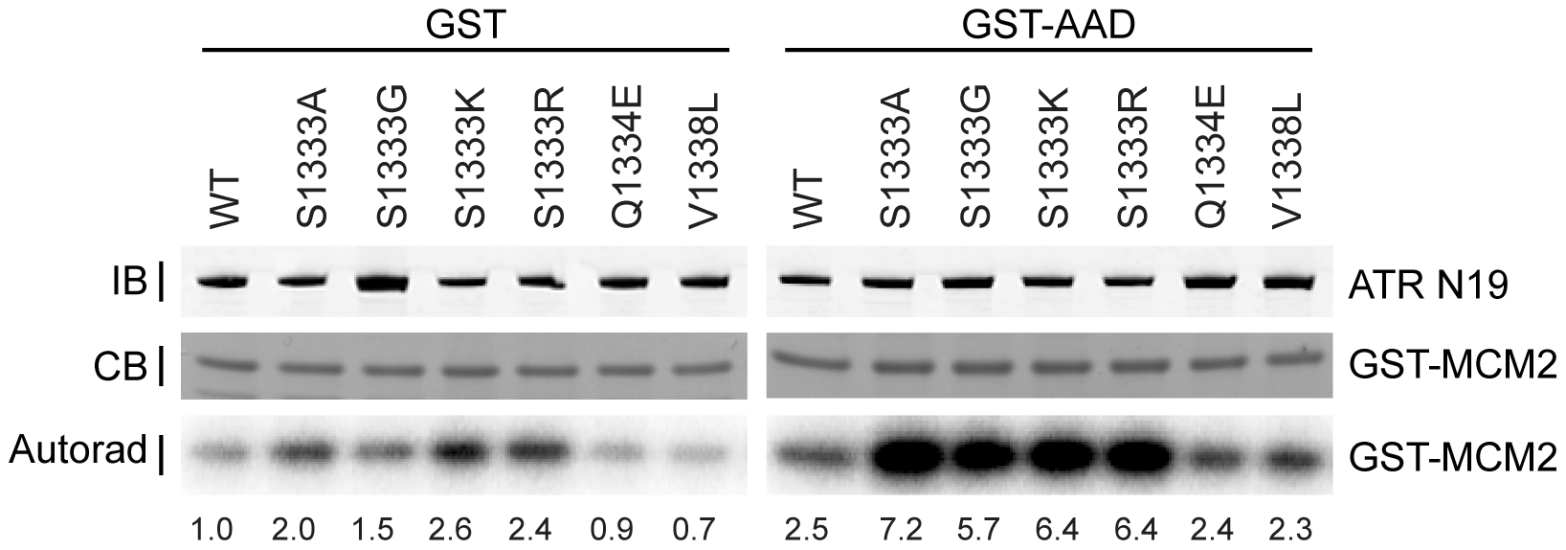
Additional S1333 mutations alter ATR kinase activity. The indicated ATR mutant or wild type proteins complexed with ATRIP were purified and incubated with GST-MCM2 substrate, [γ-^32^P]-ATP, and GST-TOPBP1-AAD (GST-AAD) or GST. Kinase reactions were separated by SDS-PAGE and [^32^P]-MCM2 detected by autoradiography. Quantitation of the fold activation compared to wild type protein incubated with GST measured by a phosphorimager is indicated below each lane. The amount of ATR and MCM2 present was detected by Coomassie blue (CB) or immunoblotting (IB) as indicated. Each mutant was tested at least three times and a representative experiment is shown.

We created additional amino acid mutations at S1333 and tested their kinase activities. First, we created an aspartic acid mutation, to mimic phosphorylation. S1333D-ATR had less kinase activity than wild type ATR upon stimulation by TOPBP1 and less activity than wild type without stimulation (Fig. 1C). Conversely, S1333A-ATR is more active than wild type ATR with or without the addition of TOPBP1. Next, we mutated S1333 to glycine, further reducing the size of the amino acid occupying this position from the alanine mutation. We also created arginine and lysine mutations to create a positive charge at this position. All of these mutations created a hyperactive kinase similar to activity levels of S1333A-ATR, with TOPBP1 (Fig. 2). They also exhibited slightly elevated kinase activities without TOPBP1 although with some variability in the magnitude (Fig. 2). Thus, all mutations of S1333 tested altered ATR kinase activity, with most increasing activity and the S1333D mutation decreasing activity.

Additionally, we tested select mutations in this ATR region identified through cancer genome sequencing efforts. Q1334E is a mutation found in colorectal cancer and V1338L was found in cancer of the pleura. Neither of these mutations changed ATR kinase activity *in vitro* (Fig. 2).

**Figure 3:**
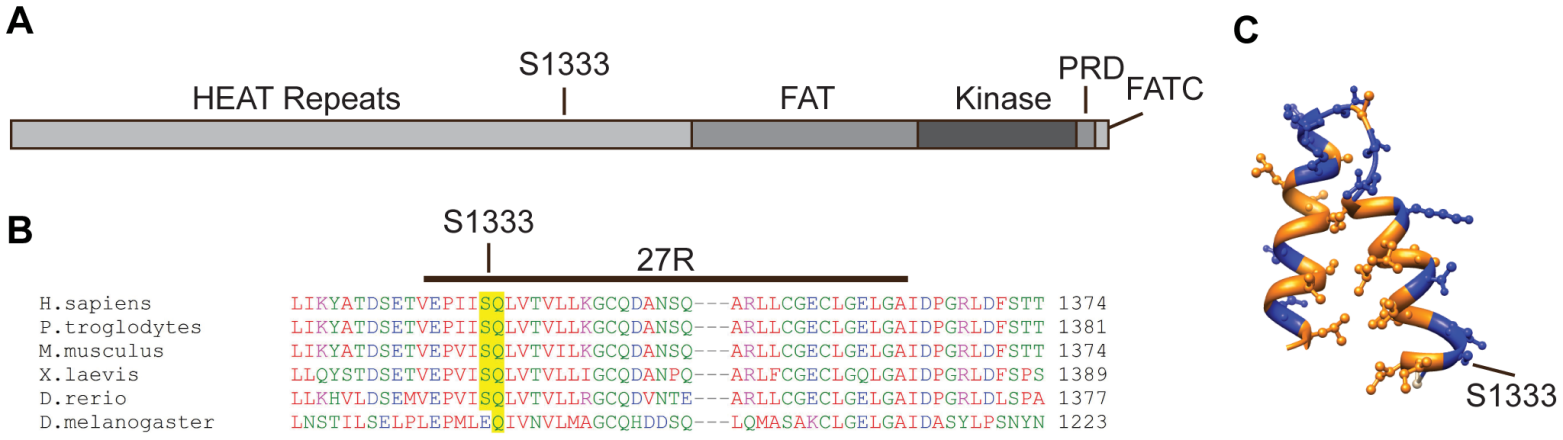
S1333 is a conserved amino acid within HEAT repeat 27 of ATR. (A) Schematic representation of the ATR protein. (B) Sequences of ATR orthologs were aligned using ClustalW2. (C) Phyre2 was used to predict the structure of HEAT repeat 27. Polar amino acids are colored blue.

ATR is a large protein containing 45 HEAT repeats [3]. S1333 is located within HEAT repeat 27 of ATR (Fig. 3A–B and [3]). A Clustal W2 sequence alignment shows conservation of S1333 in vertebrates (Fig. 3B). Using Phyre2 to predict the structure of HEAT repeat 27, S1333 is located on the predicted, polar exterior of helix one (Fig. 3C). This region of ATR has not previously been implicated in its regulation.

### S1333 is Unlikely to be Phosphorylated in Cultured Cells

Our *in vitro* data indicated that changing S1333 to a non-phosphorylateable residue activated ATR, while changing it to a phospho-mimetic decreased its activity. Since S1333 is followed by a glutamine, creating a consensus site for ATR auto-phosphorylation, we entertained the possibility that S1333 phosphorylation regulates ATR. To investigate whether S1333 is phosphorylated, we used three approaches: mass spectrometry, generation of a phospho-peptide specific antibody, and *in vitro* phosphorylation.

LC-MS-MS analysis of ATR purified from undamaged, HU, or IR treated HEK293T cells detected multiple phosphorylation sites, including T1989 [21]. However, we failed to detect a peptide with modifications to S1333 despite observing the unmodified peptide repeatedly (Supp. Fig. 1). We then tried to generate a phospho-peptide specific antibody to S1333. We immunized four rabbits and none yielded a purified antibody that recognized ATR in immunoblots or immunoprecipitation experiments (data not shown). Finally, we generated a short ATR protein fragment containing S1333 and tested whether this recombinant protein was phosphorylated on S1333 by purified ATR in an *in vitro* kinase assay. Again, we failed to detect significant S1333 phosphorylation (data not shown). Thus, while these negative data do not exclude the possibility that S1333 is phosphorylated, we do not have evidence that it is phosphorylated either in cultured human cells or during *in vitro* kinase assays.

### Generation of Cells Expressing only S1333A or S1333D-ATR

**Figure 4:**
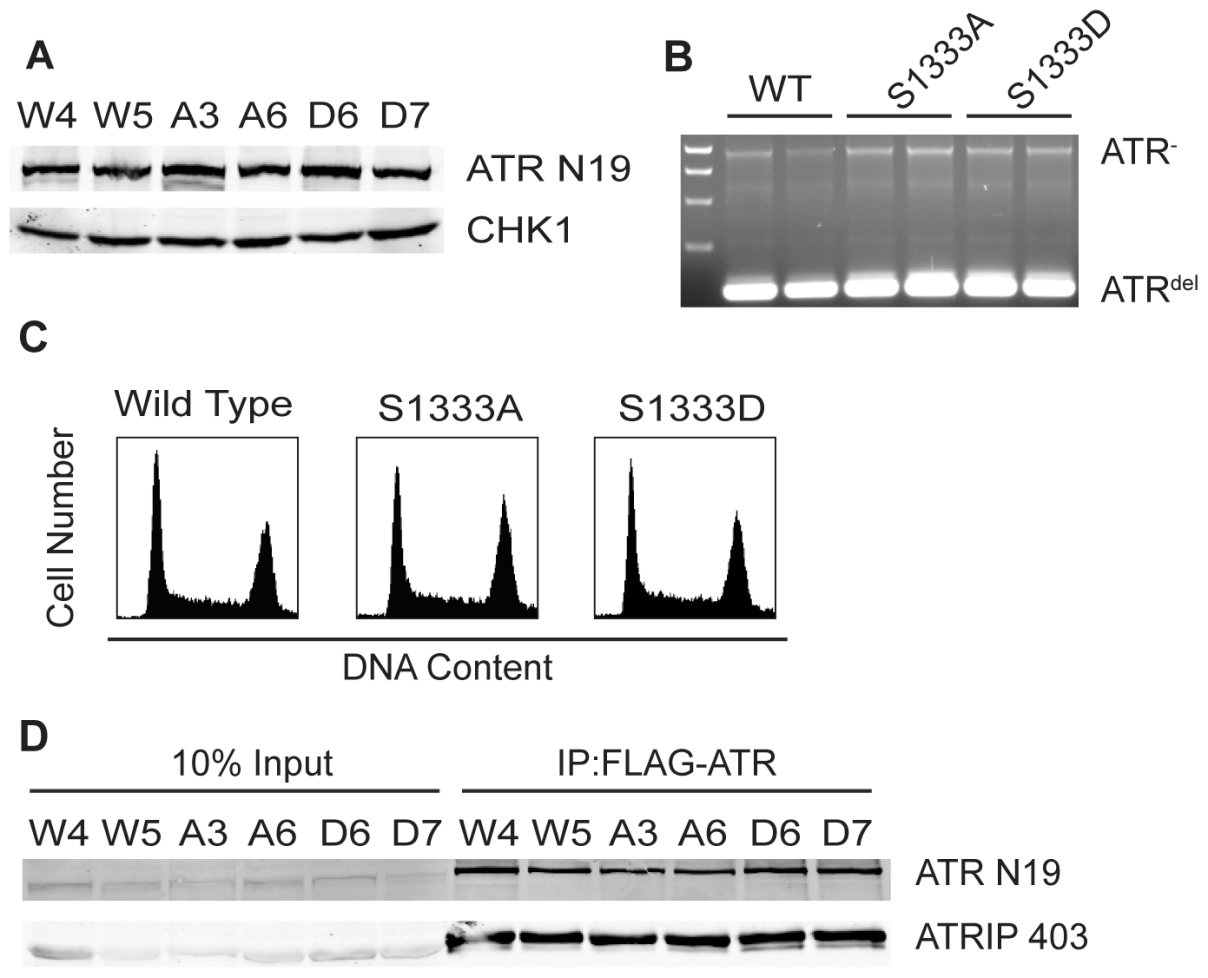
S1333A and S1333D-ATR cell lines express ATR at similar levels and maintain a normal cell cycle. ATR flox/− cells expressing wild type (WT), S1333A, or S1333D-ATR proteins were cultured in tetracycline media and infected with Cre-expressing adenovirus to delete the floxed ATR allele. Infected cells were plated at low density and several surviving colonies expanded. (A) Cell lysates were separated by SDS-PAGE and immunoblotted to determine ATR expression levels. (B) PCR genotyping confirmed deletion of the floxed allele. ATR^-^ is the PCR product derived from the neomycin-disrupted allele, ATR^del^ is the PCR product derived from the Cre excised exon 2 allele. (C) Wild type, S1333A or S1333D-ATR asynchronously growing cells were fixed, stained with propidium iodide, and examined for DNA content by flow cytometry. (D) Clonal isolates of ATR flox/− cells expressing wild type (W4 or W5), S1333A (A3 or A6), or S1333D (D6 or D7) ATR proteins were lysed, Flag-immunoprecipitated, separated by SDS-PAGE, and immunoblotted to detect the ATR-ATRIP complex.

The hyperactive S1333A-ATR protein can be a useful research tool since its increased activity, which is still regulated by TOPBP1, may facilitate *in vitro* biochemical reactions. To test if the mutant retained hyperactivity when expressed in cells and to analyze the functional consequences of mutating S1333, we utilized a genetic complementation assay using HCT116 ATR^flox/−^ cells. These cells contain one conditional ATR allele and the second allele disrupted by a neomycin cassette [21]. Additionally, the cells express the tetracycline repressor. Wild type ATR, S1333A-ATR or S1333D-ATR expression vectors, containing a tetracycline response promoter and an N-terminal FLAG-HA_3_ tag, were transfected into the ATR^flox/−^ cells. After selection, we screened stable clones for equal levels of inducible ATR. Then, we infected the cell lines with adenovirus encoding the Cre recombinase to delete the remaining intact endogenous ATR allele. The exogenous ATR protein expression was maintained with tetracycline. Stable clones were screened again for equal ATR expression and deletion of the floxed ATR allele (Fig. 4A–B). PCR genotyping to confirm Cre excision of the remaining intact ATR allele was performed as previously described [8]. Additionally, we checked for equal cell cycle distribution across the cell lines. All clones had similar distributions (Fig. 4C) and had similar population doubling times (data not shown). Additionally, all clones expressed nearly equal levels of ATRIP, which co-immunoprecipitated with the wild type and mutant ATR proteins with equal efficiencies (Figure 4D). Thus, mutation of S1333 does not alter the stability of the ATR-ATRIP complex or the growth of unperturbed cells. Multiple clonal isolates of each cell type were analyzed in all subsequent experiments to ensure results were not due to clonal variations.

### S1333A-ATR Cell Lines have Elevated Phosphorylation of ATR Substrates

**Figure 5:**
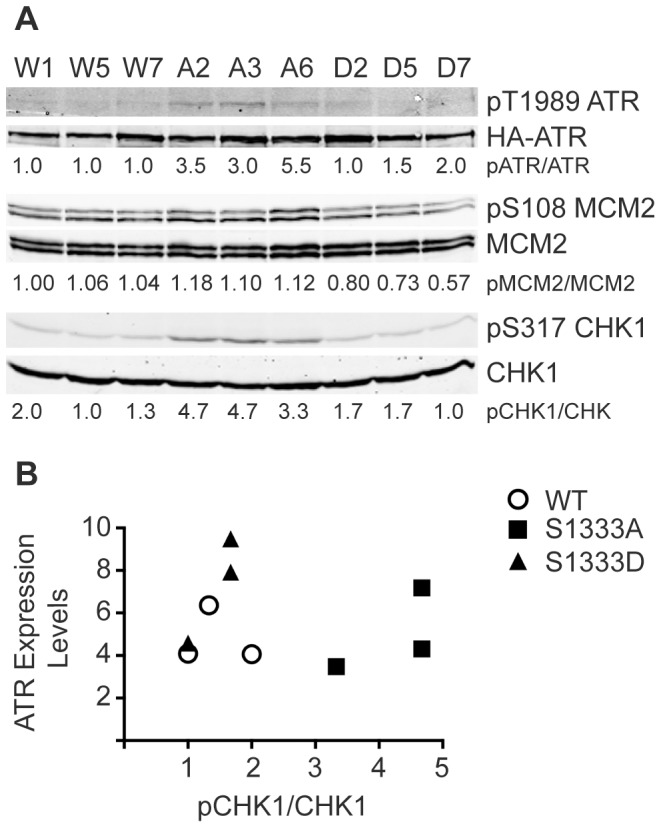
S1333A-ATR expressing cell lines contain elevated levels of phosphorylated ATR substrates. (A) Lysates from ATR−/− cell clones expressing wild type (W1, W5, W7), S1333A (A2, A3, A6), or S1333D (D2, D5, D7) ATR proteins were separated by SDS-PAGE and immunoblotted with the indicated antibodies. Quantitative immunoblotting was used and the ratio of phosphorylated protein to total protein normalized to wild type (W1) is listed below each lane. Note that three clonal isolates for each ATR protein were analyzed to ensure results were not due to clonal variation. All cell lines were examined multiple times and a representative experiment is shown. (B) The ratio of pCHK1/CHK1 and the expression levels of ATR are compared to show that the small differences in ATR expression levels in different cell lines do not account for the change in substrate phosphorylation.

*In vitro*, the basal kinase activity of S1333A-ATR is higher than wild type (Fig. 1C). To test if this is true in cells, we analyzed basal phosphorylation levels of multiple ATR substrates in three wild type, three S1333A, and three S1333D clonal cell lines without any added genotoxic stress. Phosphorylation levels were analyzed by calculating the ratio of phosphorylated protein to total protein and then normalized to wild type ATR. S1333A-ATR cells contain higher levels of phosphorylated CHK1 compared to wild type and S1333D-ATR (Fig. 5A). We also observed increased phosphorylation of ATR and MCM2 in the S1333A-ATR cell line and slightly decreased MCM2 phosphorylation in the S1333D cell line (Fig. 5A). However, we did not detect significantly decreased levels of pCHK1 and pATR in the S1333D-ATR cells. Figure 5B illustrates that the difference in pCHK1 levels in the cells is not due to small differences in ATR expression levels since there was no correlation between ATR protein expression and the pCHK1/CHK1 ratio measured by immunoblotting.

### S1333 Mutation to Aspartic Acid Causes Modest Defects in ATR Checkpoint Function

**Figure 6:**
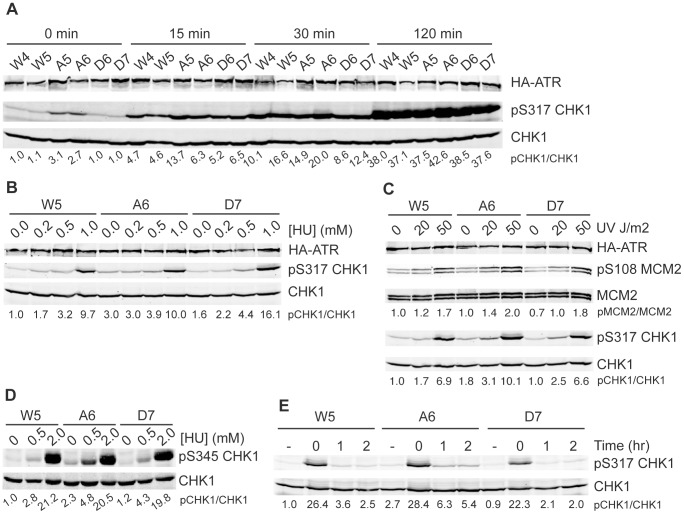
S1333A-ATR expressing cell lines maintain elevated levels of phosphorylated substrate at low replication stress levels. (A–E) Cell lysates were separated by SDS-PAGE and analyzed by quantitative immunoblotting using the indicated antibodies. Quantitative immunoblotting was used and the ratio of phosphorylated protein to total protein and then normalized to wild type is listed below each lane. (A) ATR−/− cell clones expressing wild type (W4 or W5), S1333A (A5 or A6), or S1333D (D6 or D7) ATR proteins were challenged with 2 mM HU for increasing lengths of time, (B) with increasing concentrations of HU for 4 hours, or (C) increasing doses of UV and allowed to recover for 2 hours. (D) Cells were treated with increasing doses of HU for 2 hours. (E) Cells were challenged with 2 mM HU for 2 hours and allowed to recover for 0, 1, or 2 hours. (−) indicates untreated cells. All experiments are representative images of at least two replicates.

Next, we used our mutant cell lines to study if ATR activation is perturbed in response to DNA damage. Initially, we treated cells with 2 mM HU for varying lengths of time. S1333A-ATR expressing cells had elevated basal levels of CHK1 phosphorylation as expected and the levels at early time points after HU addition were also higher than in the wild type ATR or S1333D-ATR expressing cells. However, there were no significant differences in the maximum level of CHK1 phosphorylation achieved after 2 h between S1333A, S1333D, and wild type ATR cell lines (Fig. 6A).

Next, we examined ATR signaling as a function of the amount of replication stress. We treated cells with increasing doses of HU and UV. With no treatment, pCHK1 is elevated in the S1333A-ATR cell line. At the lowest dose of UV and HU, pCHK1 levels in S1333A-ATR expressing cells continue to be elevated compared to wild type (Fig. 6B–D). This difference reduces with higher doses of HU and UV as phosphorylation becomes saturating. This same pattern is observed on an additional CHK1 phosphorylation site (Fig. 6D) and with MCM2 phosphorylation although it is not as striking since the basal level of MCM2 phosphorylation is quite high (Fig. 6C).

Finally, we monitored ATR signaling after release from HU treatment to see if the S1333 mutations alter how quickly the pathway turns off. In this recovery assay, two hours after release from HU, the wild type and S1333D lines contain slightly elevated pCHK1 compared to untreated cells. The S1333A-ATR cell lines have higher phosphorylation levels of CHK1 after recovery, but the fold difference is the same as that observed before treatment (Fig. 6E). Thus, the S1333A-ATR cell lines recover to a higher level of pCHK1 because the basal level of ATR signaling is higher. These assays did not indicate any problems with the cell lines turning off ATR signaling after replication stress.

**Figure 7:**
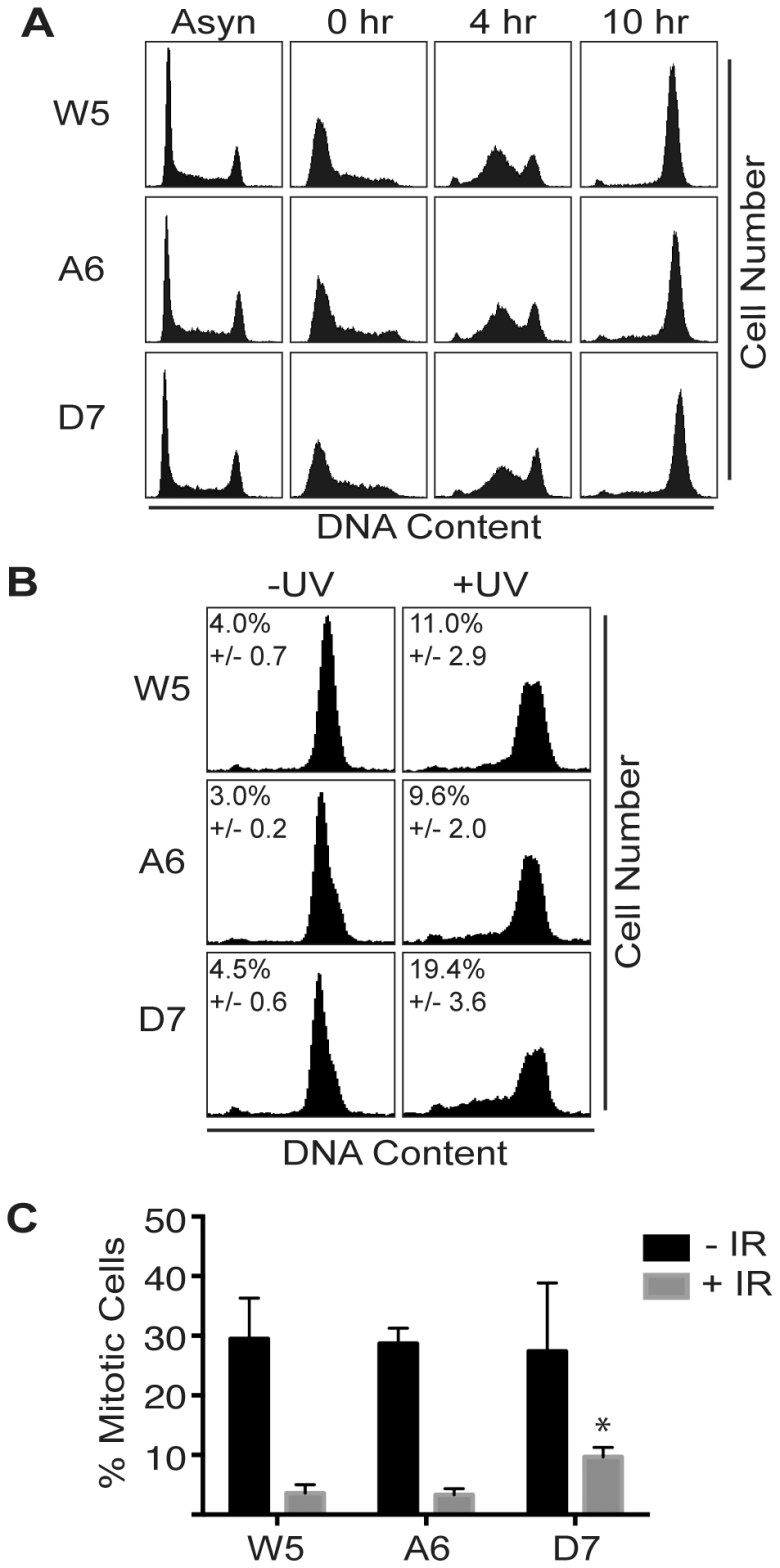
Mutation of S1333 to aspartic acid causes modest defects in completing DNA synthesis following UV radiation and in maintenance of the G_2_ checkpoint. (A–B) The indicated wild type, S1333A, or S1333D-ATR expressing cell lines were treated as indicated below, fixed, stained with propidium iodide, and DNA content analyzed by flow cytometry. (A) Cells were treated with HU for 24 hrs, released into media containing nocodazole for 0, 4, or 10 hrs, and harvested, along with asynchronously growing cells. (B) Cells were treated with or without 50 J/m^2^ UV and incubated with nocodazole for 16 hrs. The percentage of cells in S-phase was quantitated in three experiments and the mean and standard error are shown. (C) Cells were treated with or without 8 Gy IR and incubated with nocodazole for 16 hrs. The integrity of the G_2_ checkpoint was analyzed by measuring mitotic cells using flow cytometry for DNA content and phospho-specific antibody to histone H3 S10. Error bars represent the standard error of three independent experiments. The difference between IR-treated A6 and D7 cells is statistically significant (one-way Anova, p = 0.0137).

ATR is essential for completion of S-phase, recovery from replication stress, and maintaining the G_2_ checkpoint [8],[17],[30]. To test if the mutant ATR cell lines can complete S-phase following a replication challenge by HU, we treated the cells with HU for 24 hours. We then released the cells into media containing nocodazole for 0, 4, or 10 hrs. S-phase progression was monitored by flow cytometry with propidium iodide staining for DNA content. Both the S1333A and S1333D cell lines recovered and progressed through S-phase similarly to the wild type ATR cell lines (Fig. 7A). However, when the three cell lines were treated with 50 J/m^2^ UV, the S1333D-ATR cell lines had more difficulty in completing S-phase as compared to wild type or S1333A-ATR cell lines (Fig. 7B).

ATR is also needed to maintain the G_2_ checkpoint in response to ionizing radiation (IR) [8], [30]. In an initial test of this checkpoint at 6 h after IR we found no difference between wild type and S1333A-ATR cells but did see a small increase in the number of mitotic cells in the S1333D-ATR cell line although it was not statistically significant (data not shown). We repeated the assay at a longer time point and indeed found that the S1333D-ATR cells did have a modest defect in maintaining the G_2_ checkpoint in response to IR (Fig. 7C). Thus, while the hyperactive S1333A mutation alters both the *in vitro* and cellular activity of ATR, the elevated kinase activity does not alter ATR function in the S or G_2_-phase checkpoint. In contrast, the less active S1333D-ATR has sufficiently altered kinase activity to cause modest defects.

## Discussion

Our data indicate that a single amino acid change at position 1333, in a region outside of the known regulatory domains, is sufficient to alter ATR kinase activities. *In vitro* and in cells, S1333A-ATR is hyperactive compared to wild type ATR while S1333D-ATR is less active. Initially, we hypothesized this amino acid is an auto-phosphorylation site regulating ATR kinase activity. However, we were unable to obtain evidence of phosphorylation in cultured cells or in *in vitro* kinase reactions. Thus, how the mutations alter kinase activity is not clear, but we hypothesize they alter ATR structure enough to change its ability to bind substrates.

S1333 is located within the N-terminal HEAT repeats of ATR. The mechanistic role of the HEAT repeats within PIKK kinases is not known, but HEAT repeats have been shown to serve as protein-protein interaction domains and can also bind DNA [31]. In the structure of DNA-dependent protein kinase, a PIKK family member, the HEAT repeats fold into a double solenoid and form a platform on which the kinase and other C-terminal domains sit [32]. Thus, it is possible that small changes in the HEAT repeat structure are transmitted to the kinase domain, yielding a relatively large and unexpected change in activity.

ATRIP also binds to ATR through its HEAT repeats [33]. ATRIP has several functions in ATR signaling including stabilizing the ATR protein, targeting ATR to replication stress sites, and contributing to the interaction with the TOPBP1 protein [5], [8], [34], [35]. TOPBP1 binding to the ATR-ATRIP complex activates ATR by inducing an unknown structural change within ATR that increases ATR substrate affinity [19]. The mutations creating a hyperactive kinase may partly mimic the effect of TOPBP1 binding to ATR-ATRIP and potentiate the ability of TOPBP1 to promote the change in ATR conformation needed for its increased activity.

In summary, we identified single amino acid mutations within the ATR HEAT repeats that alter its kinase activity. Cells expressing S1333A-ATR have elevated basal phosphorylation levels of ATR substrates but no noticeable checkpoint or replication defects in cultured cells. Thus, cells can tolerate elevated basal ATR kinase activity. The small decrease in ATR activity caused by the S1333D mutation is enough to cause modest defects in some ATR checkpoint functions. S1333 is not in a region of ATR previously known to be involved in regulation of the kinase. Future high-resolution structural studies will aid in understanding why this region is important to regulate ATR activity levels.

## Supporting Information

Figure S1 **A peptide containing unphosphorylated S1333 can be detected by mass spectrometry.** MS/MS spectrum of ATR peptide, residues 1321–1341, is shown. The [M+3H]+3 precursor ion with m/z 773.76 was selected for fragmentation. The observed singly and doubly protonated b- and y-type product ions are assigned to their corresponding m/z peaks in the tandem mass spectrum. The amino acid sequence is provided above the annotated spectrum, and the interresidue-placed brackets denote sites of amide bond fragmentation that occurred with collision-induced dissociation (CID). b-type product ions correspond to the resulting fragment ions that contain the N-terminus of the peptide, and y-type ions correspond to C-terminal peptide fragments. Asterisks indicate product ions for which neutral loss of water has occurred.
(TIF)
