# Supplementary figures and images for: Mutation of Serine 1333 in the ATR HEAT Repeats Creates a Hyperactive Kinase
Source: PLoS One. 2014 Jun 5;9(6):e99397. doi: 10.1371/journal.pone.0099397 (PMC4047089; doi:10.1371/journal.pone.0099397)

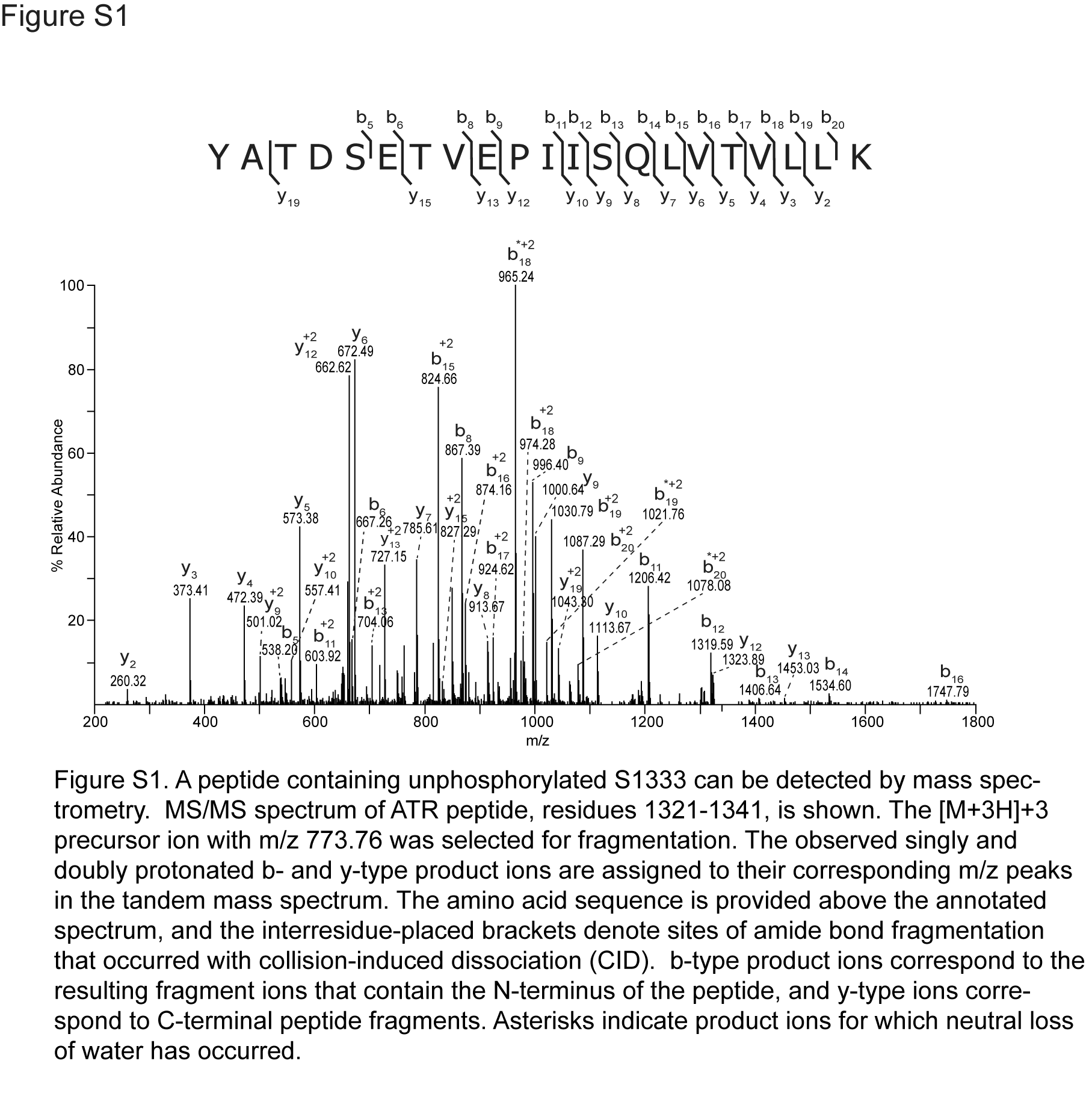

Supplement: Figure S1 — A peptide containing unphosphorylated S1333 can be detected by mass spectrometry. MS/MS spectrum of ATR peptide, residues 1321–1341, is shown. The [M+3H]+3 precursor ion with m/z 773.76 was selected for fragmentation. The observed singly and doubly protonated b- and y-type product ions are assigned to their corresponding m/z peaks in the tandem mass spectrum. The amino acid sequence is provided above the annotated spectrum, and the interresidue-placed brackets denote sites of amide bond fragmentation that occurred with collision-induced dissociation (CID). b-type product ions correspond to the resulting fragment ions that contain the N-terminus of the peptide, and y-type ions correspond to C-terminal peptide fragments. Asterisks indicate product ions for which neutral loss of water has occurred. (TIF) [file pone.0099397.s001.tif]
